# *Lactiplantibacillus plantarum* 082 ameliorates heat stress-induced testicular injury by modulating the gut microbiota

**DOI:** 10.1128/msystems.01693-25

**Published:** 2026-03-16

**Authors:** Haiyang Tu, Siyuan Shen, Dongxue Huo

**Affiliations:** 1 Key Laboratory of Tropical Fruit and Vegetable Quality and Safety of Hainan Province, College of Food Science and Engineering, Hainan University 74629, Haikou, Hainan, China; The University of Hong Kong, Hong Kong, Hong Kong

**Keywords:** heat stress, *Lactiplantibacillus plantarum*, testicular injury, short-chain fatty acids

## Abstract

Heat stress has become one of the major threats affecting animal reproductive performance. Although probiotics show potential in maintaining testicular health, their protective effects and underlying mechanisms against heat stress-induced testicular injury remain unclear. In this study, *Lactiplantibacillus plantarum* 082 (LP082) was administered to heat-stressed mice, and its role and mechanism in alleviating testicular damage were systematically evaluated through biochemical assays, histopathological analysis, gut microbiota diversity profiling, and gas chromatography-based quantification of short-chain fatty acids (SCFAs). The results showed that heat stress significantly reduced testicular weight in mice, caused testicular tissue damage, and triggered pronounced inflammatory responses and oxidative stress. LP082 intervention notably ameliorated heat stress-induced gut dysbiosis: on one hand, it significantly decreased the abundance of harmful bacteria such as *Klebsiella* and *Bacteroides*, repaired intestinal barrier damage, and effectively blocked the translocation of lipopolysaccharide (LPS) from the gut to the circulatory system, thereby reducing systemic LPS load; on the other hand, it significantly promoted the proliferation of beneficial bacteria, including *Akkermansia*, *Lactiplantibacillus*, *Bifidobacterium*, and *Faecalibaculum*, thus elevating SCFA levels. These improvements collectively mitigated systemic inflammation and oxidative stress, ultimately alleviating testicular tissue damage. In summary, LP082 exerts protective effects against heat stress-induced testicular damage via modulation of the “gut-testis axis,” providing both theoretical insights and experimental evidence for developing probiotic-based strategies to safeguard male reproductive health under high-temperature conditions.

**IMPORTANCE:**

Global warming-induced heat stress severely impairs male reproductive health, with no effective interventions available. The “gut-testis axis” is increasingly recognized but poorly studied in heat stress-related testicular injury. This study identifies *Lactiplantibacillus plantarum* 082 as a viable protector, which acts by remodeling gut microbiota, repairing intestinal barriers, and regulating lipopolysaccharide and short-chain fatty acid levels. It fills gaps in probiotic-mediated gut-testis axis regulation, provides an experimental basis for probiotic-based strategies, and offers new insights to mitigate heat stress-related reproductive harm in animals and humans.

## INTRODUCTION

As global climate warming intensifies, extreme high-temperature events are occurring more frequently, making heat stress a prominent challenge that threatens human health, agricultural production, and biological reproduction (1–3). Heat stress refers to a systemic physiological response that is triggered when the body is exposed to high ambient temperatures beyond its thermoneutral zone, leading to a disruption of thermal homeostasis (4). The testicular tissue, as a highly temperature-sensitive organ, must maintain a temperature consistently 2–7°C lower than core body temperature to support normal spermatogenesis (5). Heat stress associated with elevated temperatures poses a serious threat to the maintenance of normal testicular function. Current research indicates that heat stress can induce inflammation and oxidative stress within testicular tissue, leading to disruption of the intercellular barrier function between testicular cells (5). In addition, heat stress can activate eukaryotic initiation factor 2α subunit kinases, triggering necroptosis of spermatogonial and Sertoli cells through stress granule-mediated programmed necrosis pathways (6). The intestinal microbiota maintains a crucial symbiotic association with the host organism, thereby helping preserve systemic homeostasis. It can also remotely regulate the functions of distal organs through gut–organ axes, such as the gut–liver axes (7). Among these, the gut-testis axis represents a bidirectional signaling network connecting the intestinal microbiota, the testicular microenvironment, and spermatogenesis, where communication is mediated by microbial metabolites, immune signaling, and neuroendocrine mechanisms (8). This pathway is emerging as a promising target for developing therapeutic strategies to protect male reproductive health (8). Under heat stress, gut dysbiosis frequently occurs, which is believed to threaten host health by altering microbial composition and metabolic functions (5). For example, intestinal microbiota dysbiosis elicited by heat stress undermines spermatogenic function by perturbing the homeostasis of bile acid metabolism (9). As a category of viable microorganisms, probiotics confer beneficial impacts via regulating the intestinal microbiota, strengthening intestinal barrier integrity, and modulating host immune responses (10, 11). Moreover, they have been shown to support testicular health through multiple pathways. For example, probiotics can not only modulate the expression of key proteins in the testes and stabilize testosterone levels (12) but also enhance antioxidant capacity and regulate critical factors in the autophagy pathway, thereby restoring aberrant autophagic activity to normal (13). *Lactiplantibacillus plantarum*, a well-characterized functional bacterium, has been applied to therapeutic interventions for various metabolic disorders. This study selected *Lactiplantibacillus plantarum* 082 (LP082), a strain isolated from “Yucha,” which has been proven to possess significant probiotic potential in previous studies (14, 15). This study focused on elucidating the compound’s protective efficacy and mechanistic basis against heat stress-induced testicular impairment, with analyses centered on host physiology, gut microbiota, and associated metabolites.

## MATERIALS AND METHODS

### Animals, materials, and reagents

Male C57BL/6 mice (6 weeks) were obtained from Hunan Slac Jingda Animal Laboratory (Changsha, China). Following a 1-week acclimatization period, the mice were randomly divided into three groups according to their body weight: normal control group (CON), heat stress group (HS), and heat stress plus LP082 intervention group (no antibiotic intervention, *n* = 6). The *Lactiplantibacillus plantarum* 082, isolated from Hainan-style fermented fish tea (deposited at GDMCC under No. 61552), was cultured anaerobically in sterile de Man, Rogosa, and Sharpe broth at 37°C for 24 h. Bacterial cells in the logarithmic growth phase were then harvested by centrifugation, washed, and resuspended in phosphate-buffered saline (PBS) to a concentration of 5 × 10^9^ CFU/mL (15). Mice in the LP082 group received a daily oral gavage of 200 μL of this bacterial suspension for a total of 4 weeks, while CON and HS mice received an equal volume of PBS. After 2 weeks of the aforementioned pretreatment, heat stress was induced. For 2 weeks, the HS and LP082 groups were subjected to a specific environment (37°C, 75%–80% relative humidity), the CON group, by contrast, was maintained in conventional environmental conditions (23–25°C, 45–55% relative humidity). During this period, LP082 administration was continued in the LP082 group. Standard rodent chow for mice was obtained from Jiangsu Xietong Medical Biological Engineering Co., Ltd. Malondialdehyde (MDA) and superoxide dismutase (SOD) assay kits were purchased from Nanjing Jiancheng Bioengineering Institute. ELISA kits for tumor necrosis factor-α (TNF-α), interleukin-10 (IL-10), and interleukin-6 (IL-6) were obtained from Shanghai Xinyu Biotechnology Co., Ltd.

### Instruments and equipment

The instruments and equipment used in this study included a constant-temperature incubator (Ningbo Jiangnan Instrument Factory, China); 8890 Gas Chromatograph (Agilent Technologies, USA); CTFD-10T freeze dryer; Hettich ROTINA 32R high-speed refrigerated centrifuge (Thermo Fisher Scientific, USA); a G20 electric tissue homogenizer (Sangon Biotech Co., Ltd., China); and an Infinite 200 PRO multimode microplate reader (Tecan, Switzerland).

### Serum collection

Mice were fasted for 12 h and then euthanized by cervical dislocation following blood collection via retro-orbital puncture. Subsequently, fecal samples, cecal contents, and target tissues were collected and immediately snap-frozen in liquid nitrogen for subsequent storage. Clotting was allowed to proceed by incubating the blood samples at ambient temperature for 30 min and centrifuging them at 3,500 rpm for 15 min. The supernatant was collected and stored at –80°C for subsequent analyses.

### Measurement of oxidative stress markers and inflammatory cytokines

Testicular tissues were homogenized, and MDA and SOD levels were measured according to the manufacturer’s instructions. Separated serum samples were used to quantify the TNF-α, IL-10, IL-6, and lipopolysaccharide (LPS) levels following the protocols provided with the respective ELISA kits.

### Hematoxylin and eosin staining

Fresh tissue samples were fixed in 4% paraformaldehyde, followed by gradient dehydration, paraffin embedding, sectioning, and staining. Histopathological changes were then examined under a light microscope.

### Gut microbial diversity analysis

Upon collection (1-day pre-termination), mouse fecal samples were processed for 16S rRNA gene profiling. Briefly, total genomic DNA was isolated with a commercial kit, checked for integrity on a 0.8% agarose gel, and utilized to amplify the bacterial V3–V4 regions (forward primer [341F]: ACTCCTACGGGAGGCAGCAG; reverse primer [806R]: GGACTACHVGGGTWTCTAAT). Post-contamination control experiments were based on control validation. The resulting PCR products were purified and sequenced via the Illumina MiSeq platform. Sequencing data were analyzed using the Meiji Cloud Platform.

### SCFAs quantification

For short-chain fatty acids (SCFAs) quantification, frozen aliquots of intestinal contents were thawed and equilibrated in saturated NaCl solution for 30 min of homogenization, followed by acidification with sulfuric acid. Subsequently, the acidified mixture was treated with diethyl ether under vigorous shaking for 30 min. After centrifugation, the supernatant was transferred to GC vials for analysis. SCFAs were quantified by gas chromatography using the following parameters: Column: Agilent DB-WAX; injector temperature: 250°C; detector (FID) temperature: 250°C; carrier gas (N₂) flow rate: 1.5 mL/min; split ratio: 3:1; injection volume: 1 μL.

### Statistical analysis

Analysis of variance followed by Dunnett’s multiple comparisons test was used to assess significant differences among more than two groups. For comparisons between two groups, the Wilcoxon rank-sum test was applied. Histograms were constructed with GraphPad Prism 8.0 software, and data are expressed as mean ± standard deviation. Statistical significance was declared at *P* < 0.05, and the following asterisk notations were used: *P < 0.05, **P < 0.01, and ***P < 0.001 (version 9.0, GraphPad Software).

## RESULTS

### LP082 alleviated the inflammatory response and oxidative stress induced by heat stress

**Fig 1:**
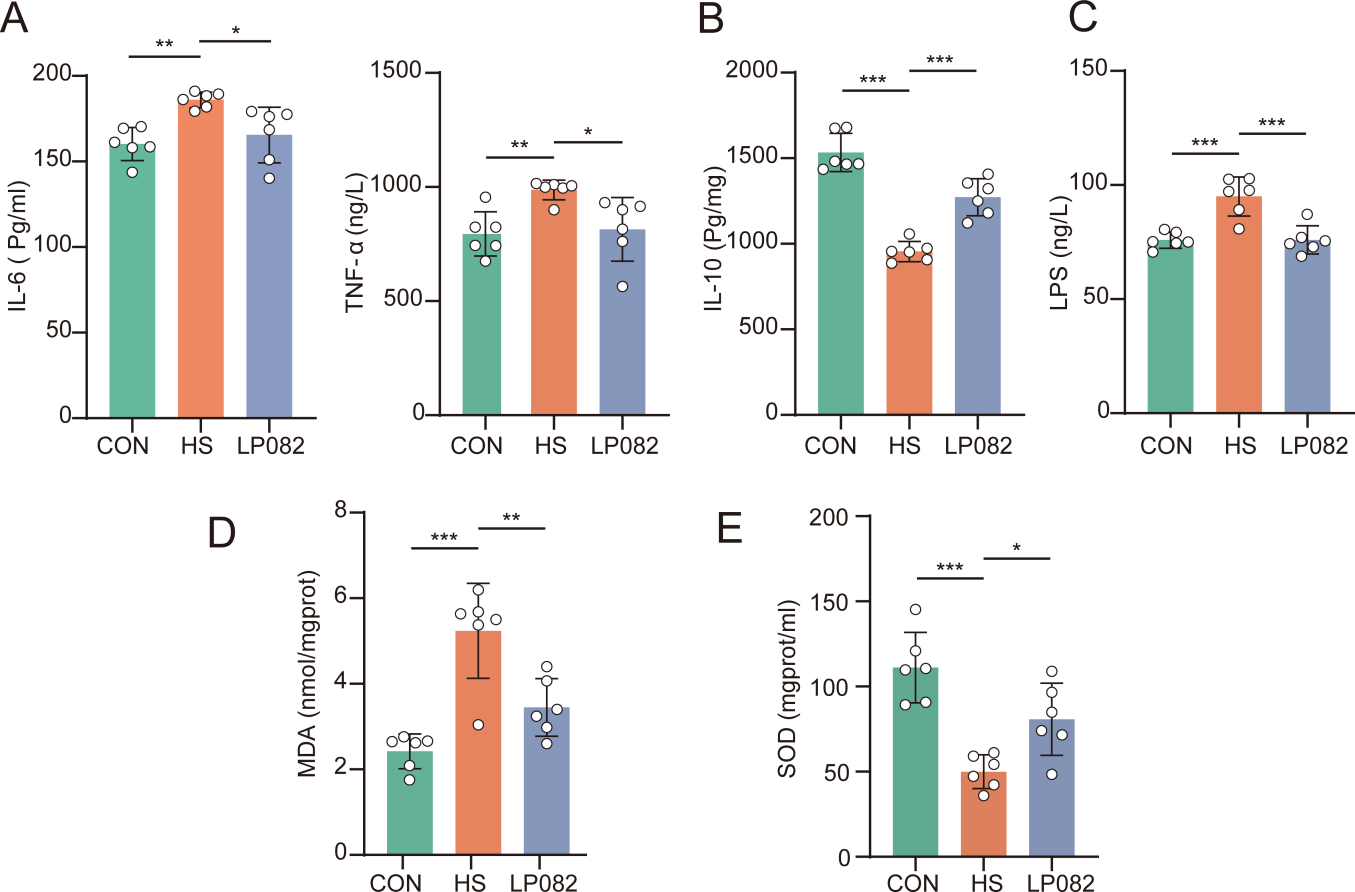
Effects of LP082 on serum parameters and testicular oxidative stress markers in heat-stressed mice (*n* = 6). (**A**) The levels of IL-6 and TNF-α. (**B**) The level of IL-10. (**C**) The level of LPS. (**D**) The level of MDA. (**E**) The level of SOD. *, *P <* 0.05; ** *P,* < 0.01; ***, *P* < 0.001.

Changes in cytokine levels are closely linked to physiological functions, immune status, disease processes, and therapy outcomes. Thus, they serve as key regulators in sustaining homeostasis and facilitating adaptation to internal and external challenges (16). In contrast to the CON group, the HS group demonstrated markedly higher levels of IL-6 and TNF-α (*P* < 0.01), coupled with a substantially lower level of IL-10 (*P* < 0.001; Fig. 1A and B). LP082 supplementation effectively mitigated the inflammatory response in heat-stressed mice, demonstrating a parallel decline in inflammatory cytokines and a notable elevation in the anti-inflammatory cytokine IL-10. As a major constituent of the outer membrane in gram-negative bacteria, LPS serves as a highly potent immunostimulant. It activates the host immune system, thereby provoking a robust inflammatory response (17). The experiment found that serum LPS levels were significantly elevated in the HS group (*P* < 0.001; Fig. 1C), and supplementation with LP082 significantly reduced serum LPS levels in heat-stressed mice. MDA and SOD are key indicators of oxidative stress in biological systems, representing the extent of oxidative damage and the capacity of antioxidant defense, respectively (18). Quantitative analysis of MDA and SOD levels revealed that in heat-stressed mice, testicular MDA levels were significantly elevated (*P* < 0.001; Fig. 1D), while SOD levels were significantly reduced (*P* < 0.001; Fig. 1E). LP082 intervention ameliorated testicular oxidative stress, evidenced by a marked reduction in MDA (*P* < 0.01) and a concomitant boost in SOD activity (*P* < 0.05). Analyses of serum and testicular tissues indicate that administration of LP082 ameliorates heat stress-induced inflammatory responses and oxidative stress damage.

### LP082 alleviated the damage of testicular and small intestine tissues induced by heat stress

**Fig 2:**
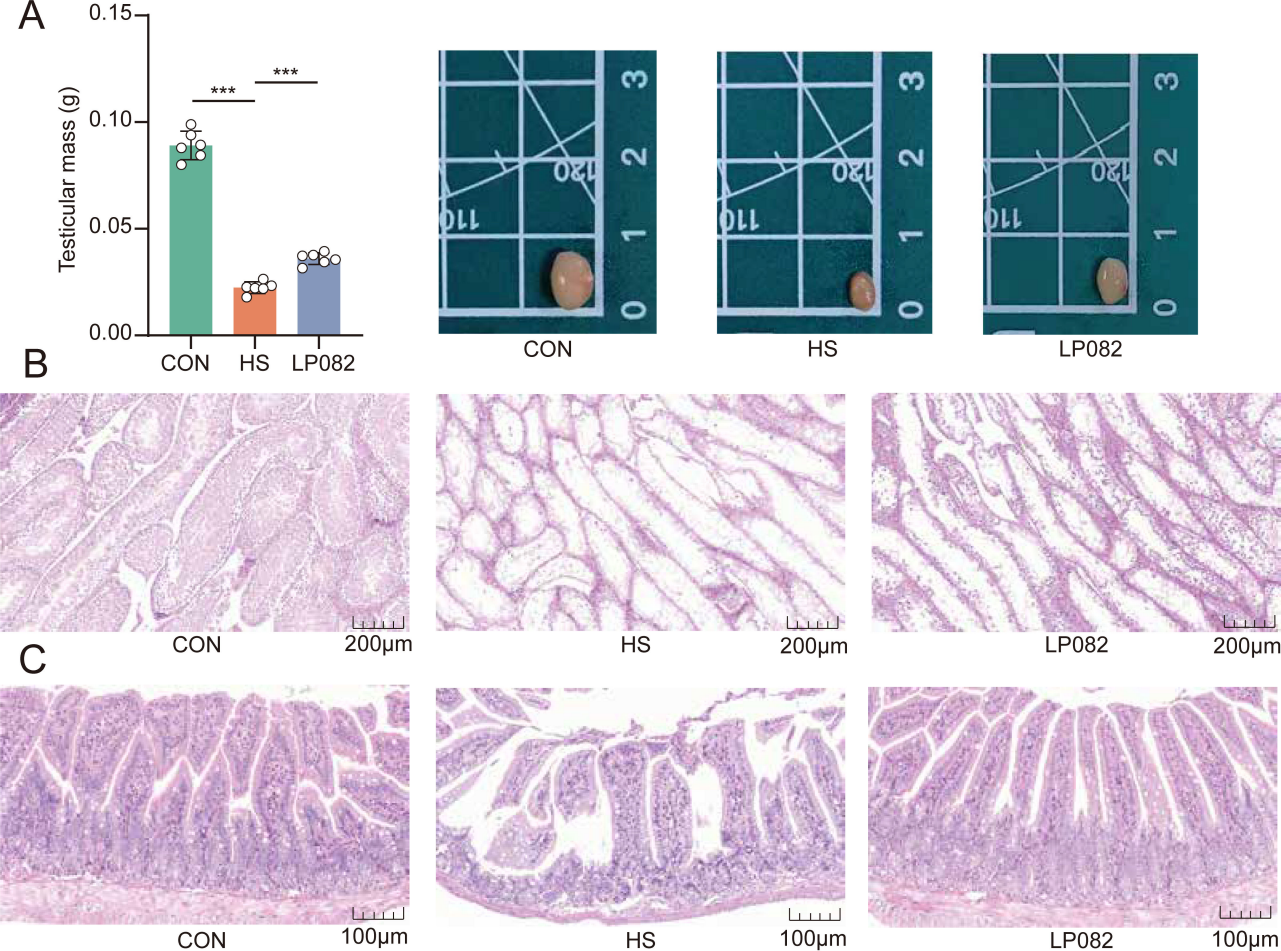
Effects of LP082 on the pathological conditions of the small intestine and testes in heat-stressed mice. (**A**) Mouse testis weight (*n* = 6). (**B**) Hematoxylin and eosin (H&E) staining of testicular tissue. Scale bar: 200 μm. (**C**) H&E staining of small intestinal tissue. Scale bar: 100 μm. *, *P* < 0.05; **, *P* < 0.01; ***, *P* < 0.001.

Testicular weight is closely associated with reproductive performance, and high ambient temperatures can significantly impair testicular physiological function, spermatogenesis, and the integrity of germ cells (19). While testicular weight was severely compromised by heat stress (*P* < 0.001), subsequent LP082 administration effectively counteracted this effect, leading to a significant recovery (*P* < 0.001; Fig. 2A). Histological analysis revealed that heat stress caused severe structural damage to both the testes and small intestine of mice. Heat stress induced seminiferous tubule atrophy, germinal epithelial sloughing, and a reduction in interstitial cells, while LP082 intervention effectively alleviated testicular damage (Fig. 2B). Under heat stress conditions, the tips of intestinal villi exhibited erosion and shedding, and the villi became atrophied, shorter, and thicker, with damaged crypts. Compared with the HS group, LP082 supplementation restored villus length to normal, with clearly visible crypt structures and markedly improved overall small intestinal architecture (Fig. 2C). Histological analysis of testicular and small intestinal tissue sections demonstrated that LP082 could, to a certain extent, alleviate heat stress-induced damage to both the gut and testes.

### LP082 ameliorated the gut microbiota dysbiosis induced by heat stress

**Fig 3:**
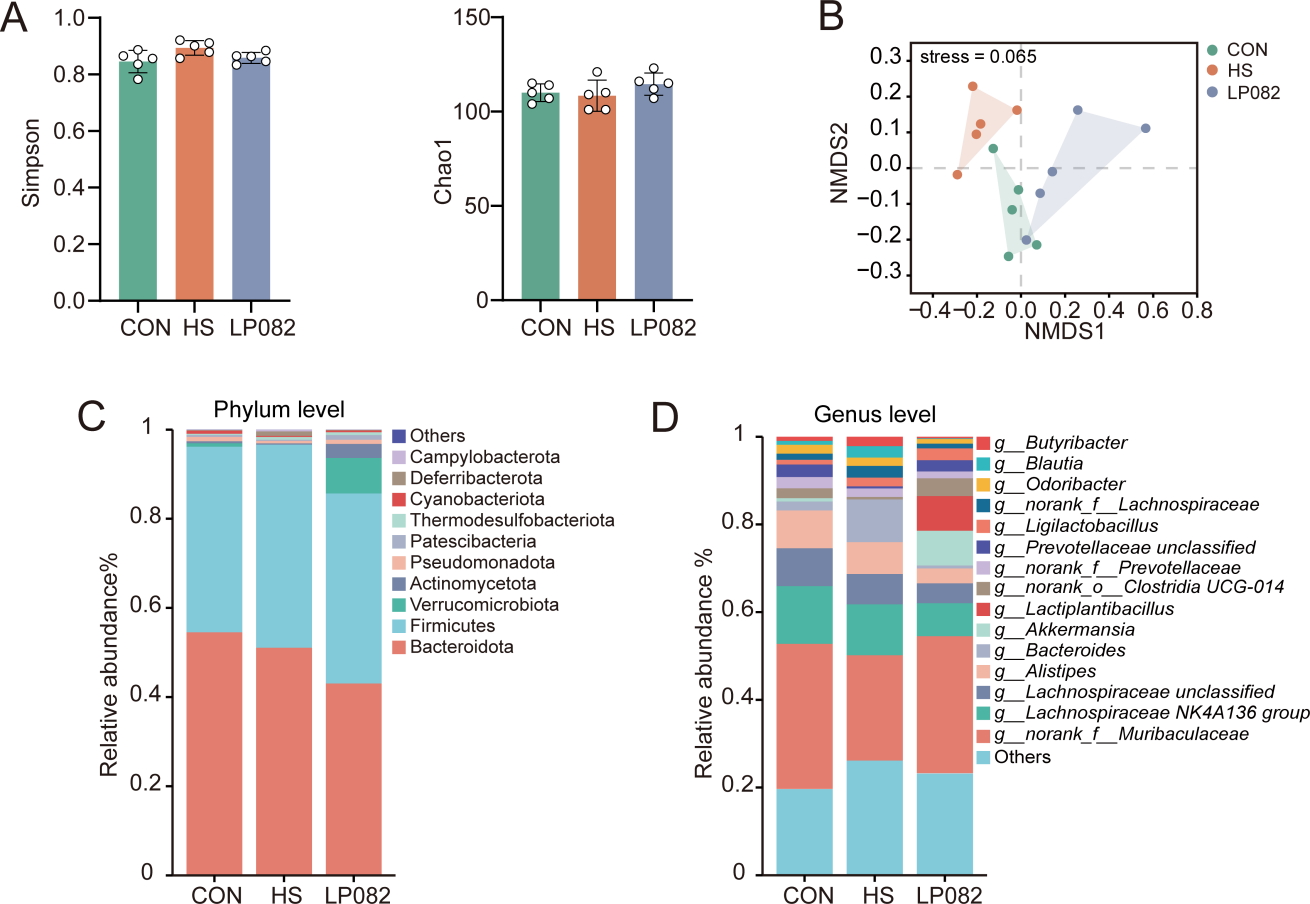
Effects of LP082 on the gut microbiota of heat-stressed mice (*n* = 5). (**A**) The Simpson and Chao1 indices. (**B**) NMDS analysis of gut microbiota. (**C**) Stacked bar plot at the phylum level. (**D**) Stacked bar plot at the genus level.

A close interconnection exists between the gut microbiota and the testes via the “gut-testis axis,” which mediates their bidirectional communication and functional interaction (8). To further characterize the gut microbial communities, we performed an analysis of their taxonomic composition. The Simpson index in the HS group exhibited an elevated trend in comparison with the CON group, and LP082 intervention ameliorated this alteration (Fig. 3A). Non-metric multidimensional scaling (NMDS) analysis revealed that the intestinal microbiota in HS group mice exhibited distinct clustering from that in the CON group, whereas the microbial community structure in the LP082 group showed a trend toward recovery, resembling that of the CON group (Fig. 3B). Analysis of the taxonomic hierarchy indicated that Bacteroidota, Firmicutes, Verrucomicrobiota, and Actinomycetota were the dominant phyla (Fig. 3C). At a finer resolution, genera including *g__norank_f__Muribaculaceae*, *Bacteroides*, *Blautia*, and *Alistipes* exhibited altered abundances. Notably, LP082 intervention partially reversed the heat stress-induced shifts in intestinal microbiota composition and augmented the abundance levels of various beneficial bacterial species (Fig. 3D).

**Fig 4:**
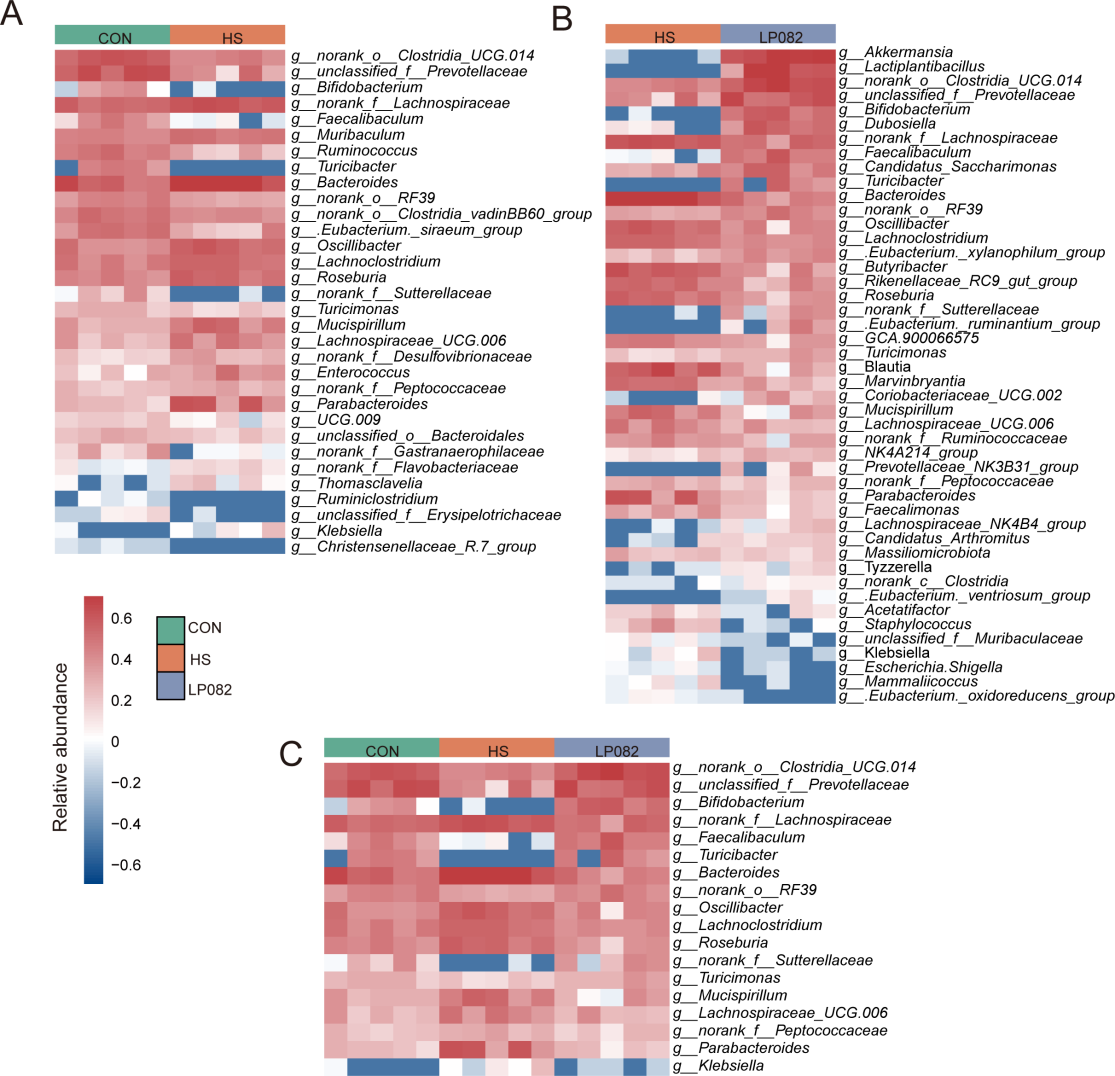
Effects of LP082 on differential bacteria (*n* = 5). (**A**) Differentially abundant microbes between the CON group and the HS group. (**B**) Differentially abundant microbes between the HS group and the LP082 group. (**C**) Microbes differentially abundant across all three groups.

Differential bacterial taxa at the genus level were further analyzed. In contrast to the CON group, the abundances of *Bifidobacterium*, *Faecalibaculum*, and *Ruminococcus* were significantly decreased in the gut of HS mice, whereas the abundances of *Enterococcus*, *Klebsiella*, and *Mucispirillum* were significantly increased (Fig. 4A). In contrast to the HS group, the abundances of *Akkermansia*, *Lactiplantibacillus*, *Bifidobacterium*, and *Faecalibaculum* were significantly increased in the LP082 group, while the abundances of *Staphylococcus*, *Klebsiella*, *Escherichia-Shigella*, and *Mucispirillum* were significantly decreased (Fig. 4B). Among these, *Turicibacter*, *Faecalibaculum*, *g__norank_f__Lachnospiraceae*, *Bifidobacterium*, *Prevotellaceae* unclassified, and *g__norank_o__Clostridia_UCG-014* exhibited significant alterations under heat stress conditions; however, their abundances were restored to near-normal levels following LP082 intervention (Fig. 4C). This indicates that heat stress disrupts the ecological balance of the intestinal microbiota, and LP082 can partially restore the microbial community structure.

### LP082 improved the functional profile of gut microbiota under heat stress

**Fig 5:**
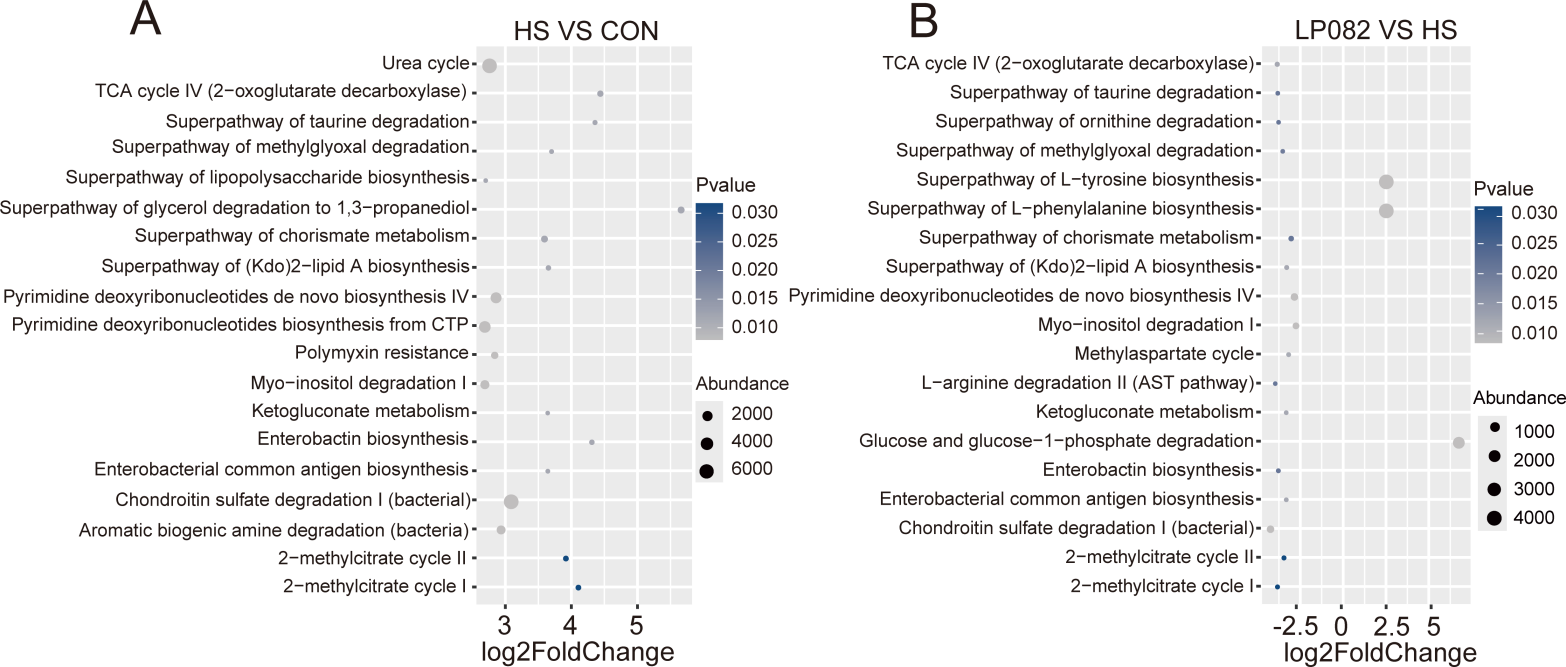
Effects of LP082 on the metabolic functions of gut microbiota in heat-stressed mice (*n* = 5). (**A**) Differentially enriched metabolic pathways between the HS group and the CON group. (**B**) Differentially enriched metabolic pathways between the LP082 group and the HS group. Differentially enriched metabolic pathways were identified using the Wilcoxon rank-sum test (*P* < 0.05). The top 19 pathways with the most significant differences were subsequently selected for further analysis based on the absolute value of the log2 fold change.

The potential functional capacities of the intestinal microbiota, predicted by PICRUSt2 and annotated using the Kyoto Encyclopedia of Genes and Genomes database, were markedly affected in HS mice. Several pathways were significantly upregulated, including those for LPS biosynthesis, (Kdo)₂-lipid A biosynthesis, polymyxin resistance, enterobactin biosynthesis, and enterobacterial common antigen biosynthesis. Notably, these pathways are characteristic of gram-negative bacteria, and their upregulation suggests that heat stress may induce a shift in the overall intestinal microbiota composition, leading to an enrichment of gram-negative taxa. In contrast, the LP082 intervention significantly downregulated key LPS-related pathways, such as the superpathway of (Kdo)₂-lipid A biosynthesis, enterobactin biosynthesis, and enterobacterial common antigen biosynthesis, while markedly upregulating superpathways involved in the biosynthesis of L-tyrosine and L-phenylalanine (Fig. 5B). These findings indicate that LPS metabolism is hyperactive in the HS group, reflecting a pro-inflammatory microbial profile. Conversely, LP082 effectively alleviates heat stress-induced metabolic dysbiosis in the gut microbiota and suppresses LPS biosynthesis, thereby contributing to the restoration of microbial and host homeostasis.

### LP082 alleviates SCFAs metabolic disorders induced by heat stress

**Fig 6:**
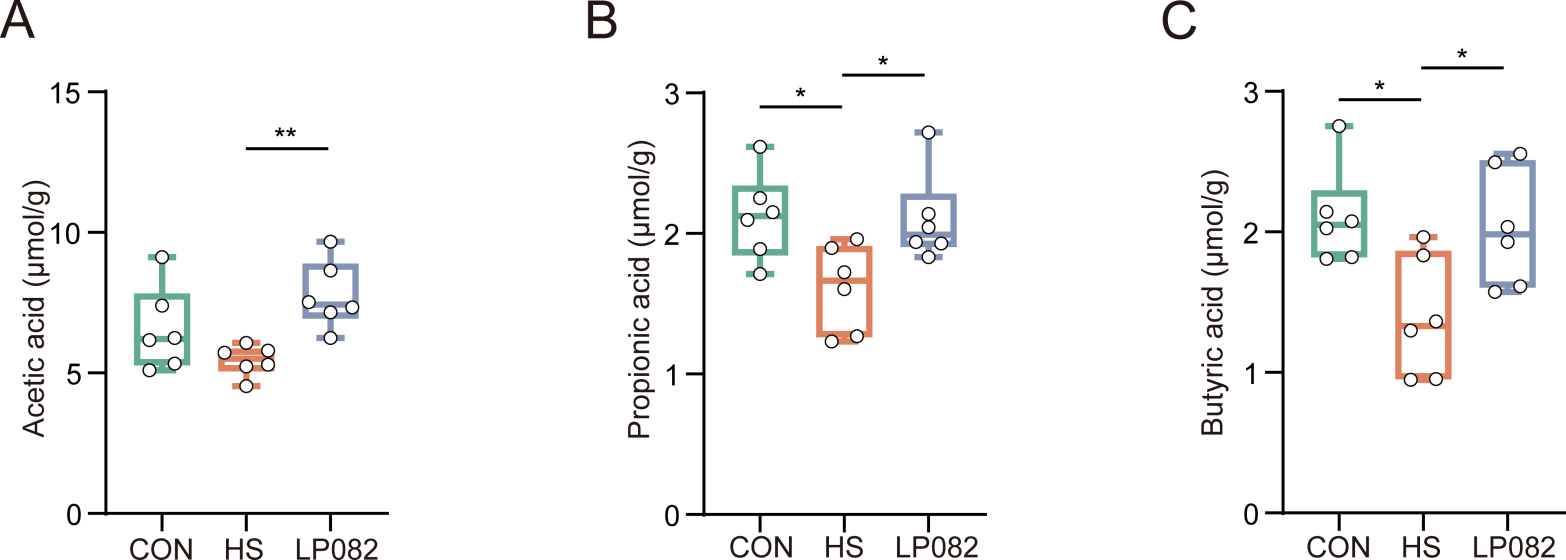
Effects of LP082 on intestinal short-chain fatty acid (SCFA) concentrations in heat-stressed mice (*n* = 6). (**A**) Acetic acid level. (**B**) Propionic acid level. (**C**) Butyric acid level. *, *P* < 0.05; **, *P* < 0.01; ***, *P* < 0.001.

As major contributors to host health, SCFAs are bioactive compounds synthesized when beneficial intestinal microbes ferment indigestible carbohydrates. They exert a pivotal role in preserving gut homeostasis, modulating immune responses, and mediating microbe–host interactions (20). As shown by gas chromatography analysis, heat stress significantly reduced the relative concentrations of propionic acid and butyric acid in the mouse gut (*P* < 0.05). Acetic acid levels also showed a decreasing trend, suggesting that the normal metabolic function of the gut microbiota may have been impaired. In contrast, the LP082 intervention significantly raised the concentrations of acetic acid (*P* < 0.01; Fig. 6A), propionic acid (*P* < 0.05; Fig. 6B), and butyric acid (*P* < 0.05; Fig. 6C) compared with the HS group. These results reveal that LP082 effectively ameliorates heat stress-induced dysregulation of SCFAs metabolism.

### Potential mechanisms by which LP082 alleviates heat stress-induced testicular injury

**Fig 7:**
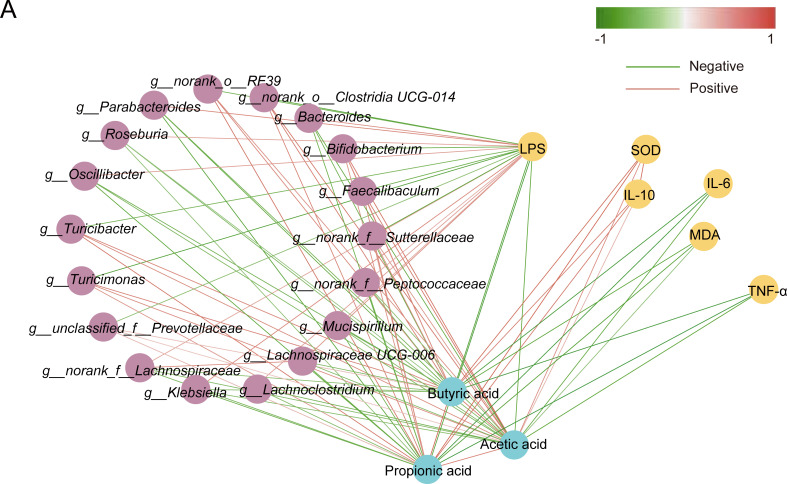
Potential mechanisms by which LP082 alleviates heat stress-induced testicular injury. (**A**) Spearman correlation network analysis. The network was constructed based on significantly correlated pairs (*P* < 0.05) with high correlation strength (|*R*| > 0.4). In the network, red edges represent positive correlations, while green edges represent negative correlations.

By investigating the crosstalk between intestinal microbiota and host metabolic processes, we sought to understand how LP082 ameliorates testicular pathophysiology and further constructed an interaction network among variables using Spearman correlation analysis(Fig. 7). Our study observed that bacterial taxa that were significantly enriched in the LP082 group, including *Turicimonas*, *g__norank_o__RF39*, *g__norank_o__Clostridia_UCG-014*, *Bifidobacterium*, and *Faecalibaculum*, exhibited a positive correlation with acetic acid, propionic acid, and butyric acid, and exhibited a negative correlation with LPS. In contrast, bacteria that significantly increased in the HS group, such as *Klebsiella*, *Mucispirillum*, and *Bacteroide*s, showed significant negative correlations with acetic acid, propionic acid, and butyric acid, and a positive correlation with LPS. Furthermore, acetic acid, propionic acid, and butyric acid were significantly negatively correlated with the pro-inflammatory cytokine TNF-α and positively correlated with the antioxidant enzyme SOD and the anti-inflammatory cytokine IL-10. These results suggest that LP082 may alleviate testicular damage by enriching SCFA-producing beneficial bacteria, thereby increasing intestinal SCFA concentrations, repairing intestinal barrier integrity, modulating systemic inflammatory responses, reducing serum LPS levels, and ultimately mitigating oxidative stress in the testes.

## DISCUSSION

**Fig 8:**
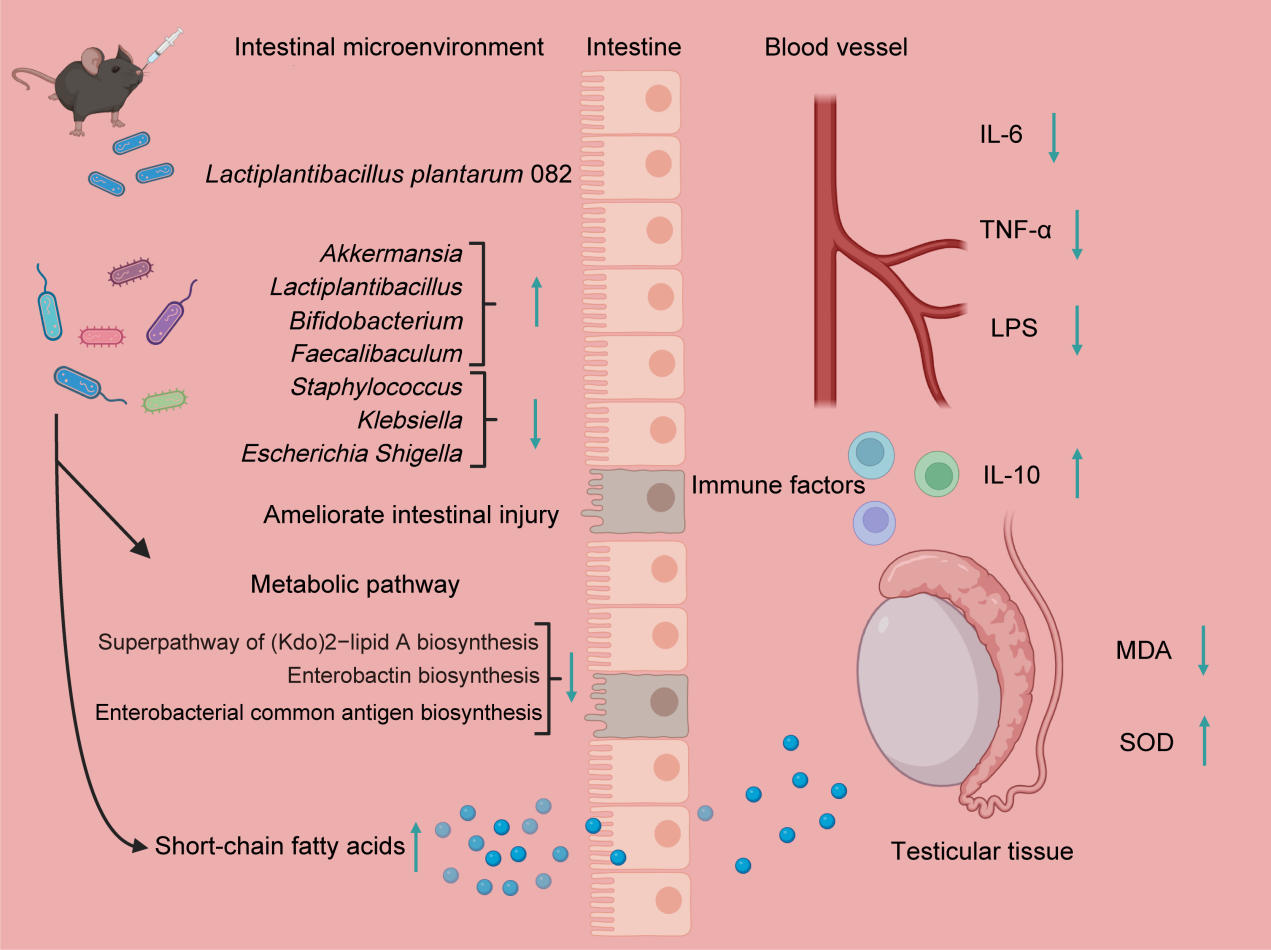
Mechanism by which LP082 alleviates testicular injury induced by heat stress.

Heat stress is a pathophysiological state that arises when the environmental temperature exceeds an organism’s thermoneutral zone or when metabolic heat production surpasses dissipation capacity, resulting in elevated core body temperature and a loss of homeostasis (1–3). This condition exerts widespread detrimental effects, with the gut serving as a primary target. Specifically, heat stress damages intestinal epithelial cells and disrupts tight junctions, leading to a compromised mucosal barrier and increased permeability (21). This heightened permeability facilitates the entry of intestinal LPS into systemic circulation, inducing a systemic inflammatory response (21). It also alters the gut microbial composition and impairs the digestion and absorption of nutrients (22). The testes are equally vulnerable due to the high thermosensitivity of spermatogenesis (5). Heat stress directly compromises the integrity of the blood-testis barrier, induces excessive apoptosis of germ cells, and disrupts the normal functions of Sertoli and Leydig cells, ultimately compromising reproductive performance in animals (19). *Lactiplantibacillus plantarum*, a lactic acid bacterium commonly isolated from traditional fermented foods such as kimchi and yogurt, as well as from natural environments (23), exhibits potential protective effects on testicular tissue. For example, *Lactiplantibacillus plantarum* TW1-1 mitigates testicular injury induced by di(2-ethylhexyl) phthalate through mechanisms involving gut microbiota modulation and inflammation reduction (24). Additionally, *Lactiplantibacillus plantarum* alleviates cadmium-induced testicular toxicity by suppressing the overexpression of inflammation-related genes, mitigating oxidative stress, and helping maintain mitochondrial DNA stability (25). Given the limited research and unclear mechanisms regarding the protective potential of *Lactiplantibacillus plantarum* against testicular injury caused by heat stress, our research constructed a mouse model of testicular injury caused by heat stress and administered LP082 intervention to investigate the therapeutic prospects and underlying pathways of *Lactiplantibacillus plantarum* in mitigating testicular injury induced by heat stress. This study revealed that heat stress provoked testicular injury in mice, as indicated by a significant reduction in testicular weight. This was accompanied by intestinal structural injury and a systemic inflammatory response, evidenced by increased serum LPS levels, enhanced production of inflammatory cytokines, and diminished production of the anti-inflammatory cytokine IL-10. Additionally, oxidative stress was apparent in the testicular tissues. Notably, LPS, a primary constituent of the gram-negative bacterial outer membrane, is liberated during bacterial death and functions as a potent immunostimulant, playing a central role in the pathogenesis of systemic inflammation, sepsis, and polysystemic failure (26, 27). Within the testes, LPS downregulates the expression of genes encoding tight junction proteins and adhesion molecules in the testes, thereby facilitating the entry of harmful substances into the seminiferous tubules and directly threatening developing germ cells (28). Gut barrier damage induced by heat stress promotes the translocation of endotoxins into the bloodstream and tissues, which may further exacerbate testicular injury. Previous studies have demonstrated that *Lactiplantibacillus plantarum* can preserve intestinal barrier integrity through synergistic mechanisms: on one hand, protection was conferred by augmenting the expression of key tight junction proteins such as ZO-1 and Occludin, thereby strengthening the physical defense; on the other hand, by inhibiting apoptotic pathways to maintain epithelial cell survival and stability (29, 30). In the present study, LP082 alleviated intestinal damage induced by heat stress, thereby reducing systemic LPS levels in mice and partially ameliorating testicular injury. It has become increasingly evident that the gut microbiota, as an integral host component, exerts profound effects on animal reproductive performance via the functional circuitry of the gut-testis axis (8). An increasing body of evidence indicates that heat stress disrupts the homeostasis of the gut microbial community. For instance, in broiler chickens, heat stress induces gut dysbiosis, marked by a decrease in beneficial bacteria such as *lactobacilli* and a concurrent increase in the proportion of potentially pathogenic bacteria like *Escherichia coli* (31). Similarly, in male dairy goats subjected to heat stress, reproductive dysfunction has been closely linked to gut microbiota dysbiosis; furthermore, fecal microbiota transplantation from heat-stressed male goats exacerbated spermatogenic impairment in recipient mice (5). Our research observed that heat stress disrupted the intestinal microbiota composition in mice, significantly reducing the beneficial bacterial abundance including *Bifidobacterium*, *Faecalibaculum*, and *Ruminococcus*, while markedly increasing the abundance of *Bacteroides*, *Enterococcus*, *Klebsiella*, and *Mucispirillum*. Notably, *Bacteroides*, *Klebsiella*, and *Mucispirillum* are typical gram-negative bacteria; their significant enrichment may contribute to elevated LPS levels in both the gut and serum. This observation aligns with the metabolic functional analysis of the gut microbiota, which revealed significant enrichment of the superpathway of lipopolysaccharide biosynthesis and the superpathway of (Kdo)₂-lipid A biosynthesis—the core bioactive component of LPS—in the gut microbiota of heat-stressed mice. Moreover, *Enterococcus* and *Klebsiella* are typical opportunistic pathogens that can cause severe infections when host immunity is compromised. *Enterococcus* is particularly notorious for its intrinsic tolerance to antibiotics and its remarkable capacity to acquire resistance, frequently leading to urinary tract infections, bacteremia, endocarditis, and intra-abdominal infections (32). *Klebsiella*, on the other hand, possesses a thick capsule that enables it to evade host immune clearance. It not only induces nosocomial pneumonia, urinary tract infections, and invasive bloodstream infections but also readily acquires and disseminates antimicrobial resistance genes, including those encoding carbapenemases (33). Additionally, *Mucispirillum* has demonstrated certain pathogenic or pro-inflammatory potential under specific conditions (34). In this study, we found that pretreatment with LP082 induced significant alterations among the gut microbial populations of heat-stressed mice, markedly reducing the abundance of gram-negative bacteria such as *Bacteroides* and *Klebsiella*. Furthermore, the superpathway of (Kdo)₂-lipid A biosynthesis was also suppressed in these mice. This suggests a corresponding decrease in the total amount of free and absorbable LPS within the intestinal lumen, thereby reducing LPS-mediated stimulation of the gut mucosa and lowering the risk of increased intestinal barrier permeability. Moreover, LP082 intervention significantly raised the relative abundance of beneficial bacterial taxa, including *Akkermansia*, *Lactiplantibacillus*, *Bifidobacterium*, and *Faecalibaculum*, offering new insights into the complex physiological changes induced by LP082 in heat-stressed mice. For instance, in a heat stress model, *Akkermansia* promotes the colonization of bile salt hydrolase-expressing bacteria, thereby modulating secondary bile acid metabolism, enhancing intestinal absorption of retinol, and subsequently elevating testicular retinoic acid levels to improve spermatogenesis (9). Similarly, *Bifidobacterium* alleviates reproductive damage caused by colitis by regulating the intestinal microbiota and enhancing microbial metabolism, which increases the abundance of ferulic acid (35). In addition to the gut microbiota itself, its metabolites play pivotal roles as mediators of host health. Among these, SCFAs, as the primary products of dietary fiber fermentation, are well recognized for their multifaceted beneficial effects, including enhancing intestinal barrier integrity, promoting microbial homeostasis, and attenuating inflammatory responses (36, 37). Current research indicates that *Lactiplantibacillus plantarum* can modulate the intestinal microbiota and increase SCFAs concentrations in the gut (38). Aligned with this, in our study, the LP082 intervention restored the levels of acetic acid, propionic acid, and butyric acid in the intestines of heat-stressed mice. This restoration of SCFAs may simultaneously ameliorate gut dysbiosis and alleviate testicular damage induced by heat stress. For instance, acetic acid has been shown to rescue diabetes-associated structural and functional abnormalities in the testes by inhibiting key regulators of cholesterol metabolism and improving insulin sensitivity (39). It also functions to restore testicular function in obese models, an effect mediated by the co-activation of both Nrf2 and PPARγ signaling pathways (40). Studies in roosters have shown that dietary butyrate supplementation improves key fertility parameters—including increased sperm motility and concentration alongside reduced abnormality rates. This is accompanied by enhanced antioxidant defenses and stimulated testosterone synthesis, collectively contributing to improved testicular development (41). In addition to their direct protective effects on testicular structure and function, SCFAs also exhibit inhibitory activity against LPS. Butyric acid significantly reduces mortality in LPS-induced septic mice (42). Acetate, propionate, and butyrate collectively exert suppressive effects on the production of pro-inflammatory cytokines and inhibit LPS-induced activation of the nuclear factor kappa B signaling pathway (43). Collectively, SCFAs hold considerable potential in mitigating testicular injury and counteracting LPS-mediated damage. In summary, this study evaluated the protective capacity of LP082 against heat stress-mediated testicular injury. The findings demonstrated that LP082 not only significantly reduced the levels of inflammatory cytokines but also markedly alleviated oxidative stress in testicular tissue, thereby ameliorating structural and functional damage induced by heat stress. Furthermore, LP082 exerted its protective effects by modulating the intestinal microbial community structure, increasing the production of SCFAs, and suppressing both the biosynthesis and intestinal translocation of LPS, ultimately preserving testicular integrity (Fig. 8).

These findings provide a promising preventive strategy for heat stress-associated testicular injury in animals and offer novel insights and practical foundations for related clinical or animal husbandry prevention and control efforts. However, this study has several limitations. First, the specific signaling pathways mediating the protective effects of LP082 remain unclear and warrant further investigation. Second, given that this is a rodent-based study, future research is needed to confirm the efficacy and safety of LP082 in livestock or human subjects.

## Data Availability

The data sets generated during the current study are available in the National Center for Biotechnology Information (NCBI) repository (PRJNA1365466). The STORMS checklist for this study is available at https://doi.org/10.5281/zenodo.18347252.
